# Genetic Diversity of Toscana Virus

**DOI:** 10.3201/eid1504.081111

**Published:** 2009-04

**Authors:** Ximena Collao, Gustavo Palacios, Sara Sanbonmatsu-Gámez, Mercedes Pérez-Ruiz, Ana I. Negredo, José-María Navarro-Marí, Marc Grandadam, Ana Maria Aransay, W. Ian Lipkin, Antonio Tenorio, María-Paz Sánchez-Seco

**Affiliations:** Columbia University, New York, New York, USA (G. Palacios, W.I. Lipkin); University Hospital “Virgen de las Nieves,” Granada, Spain (S. Sanbonmatsu-Gámez, M. Pérez-Ruiz, J.-M. Navarro-Marí); Institut de Médecine Tropicale du Service de Santé des Armées, Marseille, France (M. Grandadam); Centro de Investigacion Cooperation bioGUNE, Vizcaya, Spain (A.M. Aransay)

**Keywords:** Vector-borne infections, phlebovirus, Toscana virus, complete genome sequencing, common ancestor, dispatch

## Abstract

Distribution of Toscana virus (TOSV) is evolving with climate change, and pathogenicity may be higher in nonexposed populations outside areas of current prevalence (Mediterranean Basin). To characterize genetic diversity of TOSV, we determined the coding sequences of isolates from Spain and France. TOSV is more diverse than other well-studied phleboviruses (e.g.,Rift Valley fever virus).

*Toscana virus* (TOSV) belongs to the family *Bunyaviridae* and genus *Phlebovirus*. According to the Eighth Report of the International Committee on Taxonomy of Viruses, phleboviruses are classified into 9 serocomplexes (*1*), 1 of which includes Rift Valley fever virus (RVFV), a deadly pathogen for cattle and humans. TOSV belongs to the sandfly virus serotype Naples serocomplex. Phleboviruses have segmented RNA genomes comprised of 3 units: L (large), M (medium), and S (small). Based on the analysis of the G_N_ glycoprotein (M segment), 4 lineages of TOSV have been proposed (*2*). Moreover, phylogenetic analysis has demonstrated that TOSV isolates from Spain differ from the original isolates from Italy, TOSV strain ISS Phl.3 (*3*).

TOSV is widespread in the Mediterranean Basin, and evidence of human infection has been found in Italy, France, Spain, Portugal, Cyprus, and Turkey. The main clinical manifestation is neurologic dysfunction (*4*). Although the virus is an important pathogen (*4*), little genomic information is available for TOSV. We believe that obtaining genome sequence information for viruses with poor representation in public databases is an urgent task for the virologic community, especially for programs of virus surveillance and study of emerging pathogens. The increasing popularity of nucleic acid–based methods of detection underscores the need for a deeper knowledge of sequence variability of wild-type strains to ensure the sensitivity and specificity of the diagnostic assays. We therefore characterized the genetic diversity of TOSV by determining the coding sequences of isolates from Spain and France.

## The Study

Viruses were recovered from Vero E6 (ATCC CRL-1586) cell cultures of cerebrospinal fluid samples (CSF) from 2 TOSV-infected patients and from a pool of sandflies. The first patient was a 30-year-old man living in a rural area of Granada, Spain. He had been admitted to hospital with headache, nausea, and fever; he was found to have meningeal and CSF pleocytosis. TOSV ESH 62100 was detected in an acute-phase CSF sample by reverse transcription–PCR (RT-PCR) (*5*). The second patient was a 57-year-old woman from Augbane (Marseille area), France, who had fever, malaise, photophobia, neck rigidity, and vomiting. TOSV-specific immunoglobulin M was detected in an acute-phase serum sample; a convalescent-phase sample indicated seroconversion. Her CSF sample was positive for TOSV by PCR (*6*) and was the source for the isolation of TOSV H/IMTSSA. TOSV EsPhGR40 was isolated from a pool of female sandflies captured in the metropolitan area of Granada (*3*).

**Figure 1:**
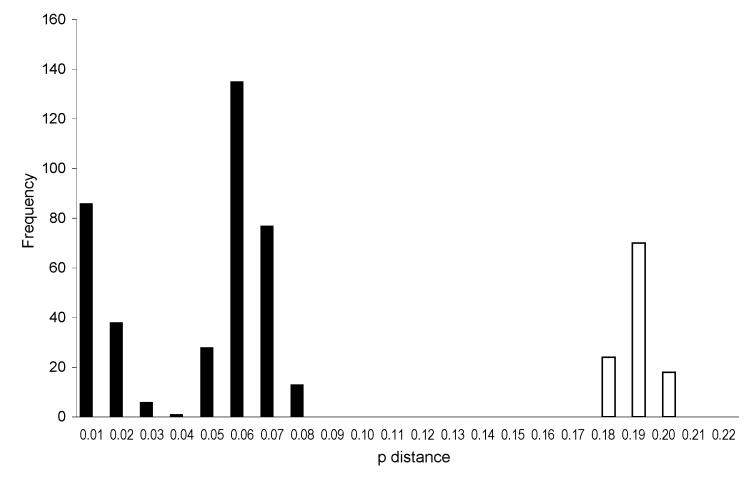
Histogram showing distribution of nucleotide pairwise (p) distances in the medium segment of Toscana virus. p distances are for nucleotides; frequencies are for intervals of 0.01. Validity of this method was confirmed by analysis of variance, comparing the scores of sequence comparisons within genotypes to those between genotypes. Black bars indicate intralineage distribution; white bars indicate interlineage distribution.

Virus sequences were obtained by using conserved primers (primer sequences available on request). GenBank accession numbers of the new TOSV sequences were EF120631 and FJ153279–FJ153286. To assess the potential for establishing a simple cutoff for classification of TOSV genotypes (e.g., similar to programs created for mumps) (*7*), we used pairwise sequence comparison to compare these sequences with all published phleboviral sequences. Sequence analysis of the M segment was sufficient to enable determination of genotypes (Figure 1). Two different clusters were clearly distinguished. We propose to name those clusters genotypes A and B. The previously described TOSV lineages that include isolates from Italy all clustered in the proposed TOSV A genotype. Comparision with RVFV, for which 7 lineages (A to G) have been described (*8*), showed higher divergence in L and M segments and nonstructural (NS) and nucleoprotein (N) genes between the proposed TOSV genotypes than that found among RVFV lineages (Table).

**Table:** Sequence differences among phleboviruses*

Comparison	Similarity of sequences (amino acid similarity), mean % ± SD			
	L segment	M segment	NS gene	N gene
Within TOSV	91.7 ± 0.7 (98.8 ± 0.5)	92.4 ± 0.6 (95.4 ± 0.9)	90.7 ± 0.8 (95.8 ± 1)	95.9 ± 0.8 (100)
Within TOSV A	NA	99.3 ± 0.1 (99.4 ± 0.5)	NA	100 (100)
Within TOSV B	98.2 ± 0.2 (99.7 ± 0.1)	95.5 ± 0.4 (98.2 ± 0.5)	97.3 ± 0.4 (98.5 ± 0.5)	98.5 ± 0.3 (100)
Between TOSV A and TOSV B	85.2 ±0.4 (97.0 ± 0.4)	81.8 ± 0.4 (90.0 ± 1.1)	83.6 ± 1.1 (89.6 ± 1.6)	87.9 ± 1.4 (100)
Within TOSV A G1 to G4	NA	99.3 ± 0.1 (99.3 ± 0.2)	NA	NA
Between TOSV A G1, G2, G3, and G4	NA	95.6 ± 0.4 (97.3 ± 0.5)	NA	NA
Within RVFV	97.7 ± 0.3 (99.8 ± 0.1)	95.4 ± 0.5 (98.6 ± 0.5)	97.2 ± 0.3 (98.5 ± 0.4)	97.6 ± 0.5 (99.8 ± 0.1)
Within RVFV lineages	98.8 ± 0.1 (99.5 ± 0.1)	98.8 ± 0.1 (99.5 ± 0.1)	98.8 ± 0.2 (99.1 ± 0.3)	99.1 ± 0.2 (99.9 ± 0.1)
Between RVFV lineages	96.6 ± 0.2 (99.2 ± 0.2)	96.6 ± 0.2 (99.1 ± 0.2)	97.0 ± 0.5 (98.0 ± 0.6)	97.5 ± 0.5 (99.6 ± 0.3)
Within SFSV	NA	76.5 ± 1.3 (80.2 ± 2.1)	99.7 ± 0.2 (98.9 ± 0.7)	100 (100)
Within SFNV	NA	72.2 ± 1.2 (72.9 ± 2.1)	NA	NA
Within PTV	96.5 ± 0.7 (100)	92.5 ± 0.8 (97.5 ± 0.9)	87.6 ± 1.4 (94.2 ± 1.6)	100 (100)
Between SFSV and PTV	NA	45.5 ± 1.8 (32.3 ± 3.2)	40.2 ± 1.9 (23.7 ± 3.1)	55.0 ± 3.6 (52.5 ± 6.4)
Between SFNV and TOSV	NA	57.2 ± 1.7 (53.6 ± 3.1)	NA	78.8 ± 2.8 (93.2 ± 3.3)

*Based on complete genome sequences. To estimate the evolutionary divergence over sequence pairs between groups, the average number of nucleotide differences per site over all sequence pairs between groups and among groups was calculated. Standard error estimates were obtained by a bootstrap procedure (500 replicates). For each pairwise sequence comparison, all positions containing alignment gaps and missing data were eliminated. L, large; M, medium; NS, nonstructural; N, nucleoprotein; TOSV, Toscana virus; NA, not applicable; RVFV, Rift Valley fever virus; SFSV, sandfly fever Sicilian virus; SFNV, sandfly fever Naples virus; PTV, Punta Toro virus.

**Figure 2:**
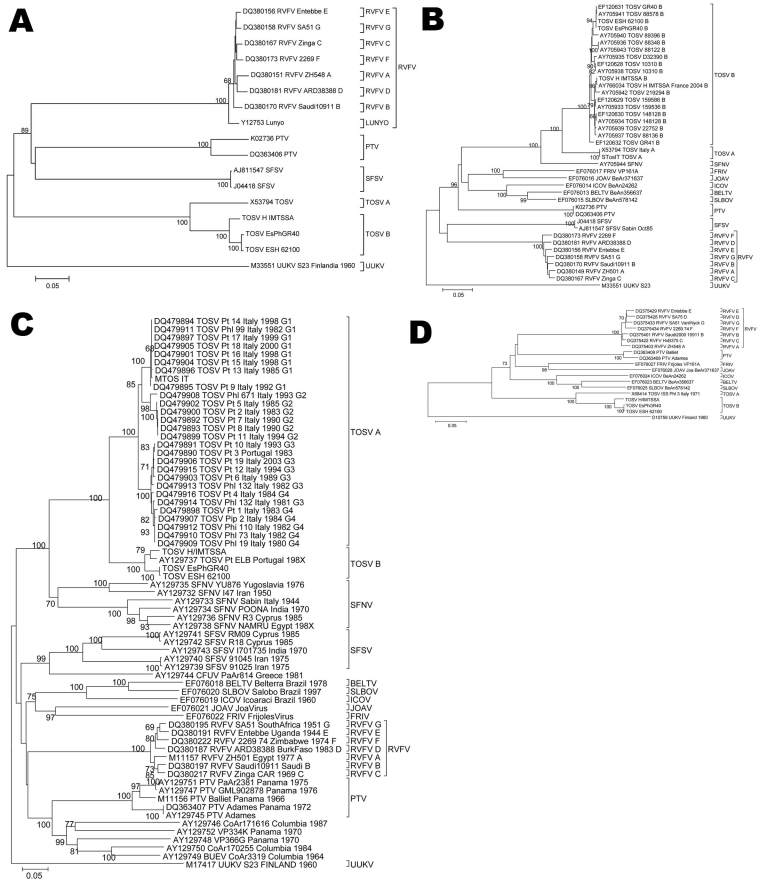
Phylogenetic analysis of Toscana virus (TOSV) strains. Coding regions for A) nonstructural (NS) gene, B) nucleoprotein (N) gene, C) medium (M) gene, and D) large (L) gene were studied by using distance MEGA (www.megasoftware.net); relationships between different strains are shown. Each sequence used shows GenBank accession number followed by name of the virus according to the International Committee on Taxonomy of Viruses and the corresponding strain. Proposed virus genotypes (TOSV from Italy, A; TOSV from Spain, B) are indicated. For TOSV, 4 lineages (G1 to G4) are shown, and for RVFV, 7 lineages (A to G) are shown, as previously described (2,8). The sequences obtained in this work are TOSVFR (TOSV H/IMTSSA), TOSVGH (TOSV ESH 62100), and TOSVGR (TOSV EsPhGR40). RVFV, Rift Valley fever virus; PTV, Punta Toro virus; SFSV, sandfly fever Sicilian virus; UUKV, Uukuniemi virus; SFNV, Sandfly fever Naples virus; FRIV, Frijoles virus; ICOV, Icoaraci virus; JOAV, Joa virus; BELTV, Belterra virus; SLBOV, Salobo virus; BUEV, Buenaventura virus. Scale bars indicate nucleotide substitutions per site.

Pairwise comparison demonstrated the following: 1) all TOSV S segments were highly conserved; 2) the L segment demonstrated less conservation than the N gene at the nucleotide or deduced amino acid levels; 3) TOSV M segments were the most divergent; and 4) variation in M segments was higher than that among RVFV strains but less pronounced than that within the group of sandfly fever Sicilian viruses or sandfly fever Naples viruses (Table). With regard to the conserved S segments, the NS gene was more variable than the N gene, and despite a nucleotide pairwise difference of 12.1% in the N coding region, the amino acid sequences were completely conserved (100% identity);

Phylogenetic analysis of all phlebovirus sequences was performed by using the maximum-likelihood method available in PAUP* under tree bisection and reconnection branch swapping; the best-fit model of nucleotide substitution in each instance was determined by using MODELTEST (*9*) (parameter values available upon request) (Figure 2). Time to most recent common ancestor (TMRCA) was estimated for the M segment by using the data from 32 dated samples collected over 35 years and the Bayesian Markov Chain Monte Carlo approach (BEAST package; *10*). We applied a relaxed molecular clock with an uncorrelated exponential distribution of rates, a general time reversible + I + F4 model of nucleotide substitution and logistic population growth. Only the M segment had enough sequence representation to perform the analysis. Nonetheless, because glycoproteins are present on the surface of virions, they are the proteins most exposed to the selective pressures of the host.

The estimated mean rate of evolutionary change was 9.1 × 10^–5^ substitutions/site/year (95% highest probability density [HPD] = 2.5 × 10^–4^ to 2.7 × 10^–6^ substitutions/site/year). Under this rate the mean TMRCA was estimated to be 3,265 years, although with wide variance (178–11,000 years). Studies of RVFV, a closely related phlebovirus (*11*) have estimated the mean RVFV M segment rate to be 2.42 × 10^–4^ (95% HPD = 1.8 × 10^–4^ to 3.0 × 10^–4^ substitutions/site/year) (*8*). TOSV M segment TMRCA is higher than RVFV M segment TMRCA (117.3 years [variance 95–143 years]) (*8*). Although the wide variance of the TMRCA calculations might be affected by the scarce sequence information available, the differences observed might also be related to their biological differences. Both are phleboviruses transmitted by the bite of arthropods that could also be implied as amplifier hosts; however, because TOSV is believed not to be amplified on its mammalian hosts, it seems that the RVFV cycle includes mammals. The high conservation of RVFV genome sequences has been interpreted as indicating that the overall tolerance for mutation within the RVFV genome is low or that the viruses in the group have a relatively recent common ancestor (*12*). More studies are needed to understand the significance of the high degree of purifying selection observed in TOSV.

## Conclusions

Our main goal, to improve the knowledge of sequence information for this neglected genus, was achieved by the addition of 3 full genome sequences of TOSV. Geographic distribution may be different for each TOSV genotype. Whereas the TOSV A genotype circulates in Italy, France, and Portugal, the TOSV B genotype circulates in Spain, Portugal, and France. Both genotypes have been reported previously in France (*13*); however, the cocirculation of both genotypes in Portugal is confusing because sequences obtained independently in 2 laboratories from strains allegedly obtained from the same patient clustered in different clades (DQ479890 and AY129737) (*14*,*15*). Geographic differences in genotype distribution may relate to differences in vector distribution.

The distribution of phlebotomines is evolving with climate change and has implications for the epidemiology of vector-borne infectious diseases. Preexisting immunity almost certainly plays a role in limiting illness in the Mediterranean Basin; pathogenicity may be higher in naive populations outside areas of current TOSV prevalence. Thus, TOSV might be considered a threat for human health, and research and surveillance programs should aim to prevent its spread to new areas.
